# Long-term patient-reported knee-related outcomes and quality of life of patients with tibial plateau fractures: A mixed-methods study

**DOI:** 10.1371/journal.pone.0356057

**Published:** 2026-08-28

**Authors:** Nynke van der Gaast, Britt H. J. Edwards, Dagmar S. Alderlieste, Ruurd L. Jaarsma, Job N. Doornberg, Michael J. R. Edwards, Erik Hermans

**Affiliations:** 1 Department of Surgery, Rijnstate Hospital, Arnhem, the Netherlands; 2 Department of Trauma Surgery, Radboud University Medical Center, Nijmegen, the Netherlands; 3 Department of Orthopaedic and Trauma Surgery, Flinders University, Flinders Medical Center, Adelaide, Australia; 4 Department of Orthopaedic Surgery, University Medical Center Groningen, Groningen, the Netherlands; University Hospital Zurich, SWITZERLAND

## Abstract

**Objective:**

This study aims to provide a comprehensive assessment of the long-term functional outcomes in patients who sustained a tibial plateau fracture by combining patient-reported outcomes and a qualitative analysis of their healthcare experiences and compare the long-term outcomes of tibial plateau fracture patients with a matched healthy population.

**Methods:**

Patients who were treated for a tibial plateau fracture at an Australian trauma-center, were asked to complete two questionnaires. The outcomes of our patient sample were compared to those of a matched sample of the general population. Lastly, in-depth interviews were conducted in a subset of participating patients to gain insight in their treatment experiences.

**Results:**

Among 165 eligible patients treated at an Australian trauma center for a tibial plateau fracture, 46 participated in the study, with 18 patients consenting to in-depth interviews. Patients who sustained a tibial plateau fracture exhibited significant worse outcomes compared to a matched healthy population. Their quality of life were significantly lower for patients with tibial plateau fractures compared to a matched health population. The qualitative analysis highlights patient perspectives and suggests potential improvements, such as standard referral to physical therapy and optimization of support services.

**Conclusion:**

This study demonstrates the substantial impact of tibial plateau fractures on knee-related outcomes and quality of life for patients, 5–10 years post-injury. The qualitative findings of this study revealed that patients wished to improve the implementation of physical therapy and improving support services to potentially achieve optimal healthcare.

## Introduction

Tibial plateau fractures are complex, intra-articular fractures. They can be caused by either high- or low-energy trauma, comprising between 1–2% of all adult fractures [1]. While high-energy trauma can lead to increasingly complex fracture patterns, in elderly individuals, low-energy injuries can also result in complex fracture patterns due to osteoporosis [2,3]. The primary treatment goal is to anatomically restore the joint surface to limit the risk of post-traumatic osteoarthritis (PTOA). The incidence of PTOA ranges from 23–44% on radiological images, with 15% of patients with severe PTOA requiring additional surgery related to their injury [4–6]. The high incidence of PTOA could adversely affect the patient’s daily functioning and participation in society within this patient group [4].

Various quantitative patient-reported outcomes (PROMs) are used across different medical disciplines to monitor the progression of a disease and/or evaluate the treatment effectiveness. Examples such as the SF-36 [7] and EQ-5D-5L [8] assess the overall quality of life. Specific PROMs are utilized for the quantification of knee symptoms; i.e., the Hospital for Special Surgery (HSS) score [9], the Knee Society Score (KSS) [10] the Rasmussen knee score [11] and the Knee injury and Osteoarthritis Outcome Score (KOOS) questionnaire [12]. Existing literature describes varying long-term outcomes; PROMs continue to improve significantly up until 1–7 years after suffering from a tibial plateau fracture [5,13–15].

While numerous studies utilize generally accepted PROMs to comprehend long-term outcomes for patients, there are various factors that can influence the recovery after fractures, which are not captured by these PROMs. Although medical professionals deemed their injuries to be “healed,” patients reported incomplete recovery and a delay in resuming their pre-injury routines. Therefore, the introduction of a qualitative analysis has been proposed to gain a more comprehensive understanding of the patient perspective following orthopaedic trauma [16–19]. The study of Rees et al. [18] aimed to enhance comprehension of patients’ experiences during their recovery 2–4 years after sustaining an open fracture of the lower limb. They identified that the concepts of vulnerability and endurance, which are clearly present in the acute setting after suffering from a traumatic fracture, continue to play a significant role in the long-term recovery process for patients as they are working on processing their functional loss and integrating these changes into their lives. To improve mental health, function, pain management and overall quality of life for patients after major trauma, this study highlights the need for extended clinical support.

To the best of the authors’ knowledge, none of the previous studies have incorporated qualitative assessments to comprehensively analyze patient experiences after sustaining a fracture. Therefore, this study aims to 1) provide a thorough assessment of the long-term functional outcomes of patients who sustained a tibial plateau fracture by combining patient-reported outcomes and a qualitative analysis of their experiences during their hospital stay and the follow-up period and 2) compare the long-term outcomes of patients who sustained a tibial plateau fracture to a matched healthy population.

## Methods

This study was approved by the hospitals’ local ethics committee (Southern Adelaide Local Health Network LNR/22/SAC/174). This study adheres to the principles of the Declaration of Helsinki (64th, October 2013).

### Subject selection

**Fig 1:**
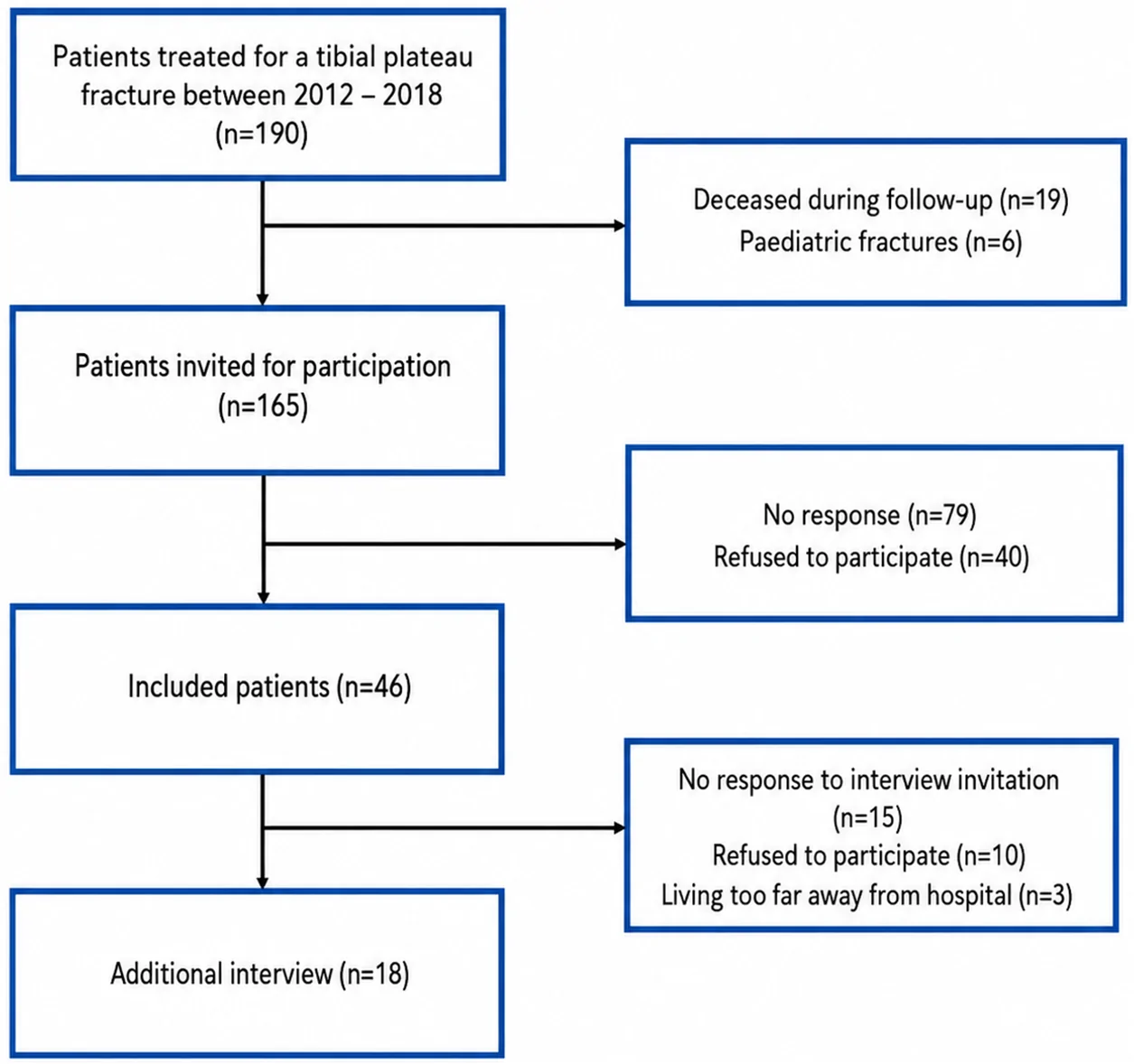
Subject selection.

Patients who sustained a tibial plateau fracture between January 2012 and January 2018 and were treated at a level I trauma center in Australia, were considered eligible for this study. Patients were identified using the hospitals’ clinical database and the hospitals’ surgical register. After identifying eligible patients, all patients <18 years old at the time of the injury and patients who died during follow-up were excluded (Fig 1: Subject selection).

### Quantitative analysis

All eligible patients were sent an invitation letter between the 1^st^ of December and the 1^st^ of March 2023 with an information folder, informed consent form and two questionnaires: the KOOS (Knee injury and Osteoarthritis Outcome Score [12]) and the EQ-5D-5L (European Quality of Life – Five Dimensions – Five Levels [20]) questionnaire. All participants provided written informed consent prior to participation in the study. The KOOS questionnaire consists of five categories: symptoms, pain, function in daily living (ADL), function in sport and recreation (Sport/Rec) and knee-related quality of life (QoL). Each category has multiple questions rated on a Likert scale ranging from 0 (no problems) to 4 (extreme problems). A sum of all questions in each category is calculated creating a score between 0 (no problems) – 100 (extreme problems). The EQ-5D-5L is used to assess general health-related quality of life in five dimensions: mobility, self-care, usual activities, pain/discomfort, and anxiety/depression. Each dimension is rated on a scale of 1 (no problems) to 5 (extreme problems), The index value scores of the EQ-5D-5L were calculated using a value set for the Australian population [21–24]. This index value is ranging from −0.5 to 1, where 1 represents perfect health, 0 indicating death and negative values indicating health states worse than death. As a separate component from the index value, the EQ-5D-5L also collects an Overall Health Score ranging from 0 (representing the worst imaginable health) to 100 (representing the best imaginable health).

Lastly, KOOS and EQ-5D-5L outcomes from a general population were obtained from published normative datasets stratified by age and gender [24,25]. Based on these strata, a group-level matched reference population was constructed by aligning our patient cohort with the corresponding age- and sex-specific categories. This approach enabled comparison of our study population with an age- and sex-comparable general population without reported knee ailments [24,25].

### Qualitative assessment

Based on the results of the returned questionnaires, the authors were interested to investigate what could cause the difference in outcomes compared to the healthy population. Therefore, a sample of the participants were asked for an additional interview to assess their hospital experiences after their knee fracture during their hospital stay and the follow-up period. Data were collected utilizing semi-structured interviews between the 15^th^ of January and the 1^st^ of March 2023, using an interview guide (S1 Appendix). The interview guide covered the following areas:

(In)hospital experiences
Expectations regarding decision making and treatment goals
Experiences regarding follow-up (i.e., clinical visits, rehabilitation, self-care and physical therapy)
Recommendations regarding treatment and follow-up

We anticipated reaching data saturation after conducting twelve interviews. Because the initial interview cohort was older than the overall study population (mean age 66.2 vs. 52.8 years), six additional interviews were conducted to include younger participants and better capture potentially different experiences among working-age patients, resulting in a total of eighteen interviews. The interviews were conducted by one researcher (NG), while a second researcher simultaneously transcribed the interview in real time. After completion, all transcripts were reviewed, verified, and refined for accuracy by the research team. The interviews were analyzed using qualitative content analysis [17]. The transcription of the interviews were optimized by the first author (NG) and two other authors (BE, DA) and they reached a consensus on the final categories and subcategories.

### Statistical analysis

SPSS version 28.0.1. was used to conduct all analyses for this study. Patient characteristics were presented as frequencies and percentages for categorical variables and as means and standard deviations for continuous variables. Continuous data was presented as a mean with standard deviations. The continuous variables of both groups were compared to each other using an independent samples t-test.

## Results

### Patient demographics

We identified 190 patients who were treated for a tibial plateau fracture between January 2012 and January 2018. 25 patients were excluded as these patients were deceased (n = 19) or were younger than 18 years at time of injury (n = 6). In total, 165 eligible patients were invited to participate in this study. In total, 46 patients participated, which resulted in a response rate of 28% (Fig 1). There were no significant differences between the non-responders (n = 119) and our participants (n = 46) in terms of age and gender. 18 patients agreed upon an additional clinical visit to create an in-depth overview of the patient experiences after suffering from a tibial plateau fracture. The mean age was 52.8 years, ranging from 18–72 years. Three of our included patients received a TKR (6,5%), Lastly, Patient demographics of all participants and the interviewed sample are represented in Table 1.

**Table 1:** Patient demographics.

Variable	Participants (n = 46)	Interviews (n = 18)	P value
**Age at time of trauma (yr)**	52.8 ± 10.2 (18–72)	51.0 ± 14.8 (18-72)	0.52
Follow-up time*	7.9 ± 1.9	7.4 ± 1.9	0.45
**Sex** Male Female	43.5% (20) 56.5% (26)	33.3% (6) 66.7% (12)	0.46
Side* Right Left	47.8% (22) 52.2% (24)	50.0% (9) 50.0% (9)	0.96
**Multitrauma** Yes No Missing	21.7% (10) 76.1% (35) 2.2% (1)	15.8% (3) 78.9% (15) 0% (0)	0.875
**Fracture pattern** Unicondylar Bicondylar	39.1% (18) 60.9% (28)	50% (9) 50% (9)	0.3
**Primary treatment** Conservative External fixation ORIF	26.1% (12) 13.0% (6) 60.9% (28)	27.8% (5) 16.7% (3) 55.6% (10)	0.80
**Additional operations** No ORIF after ex-fix TKR Removal of ORIF	83.3% (15) 13.0% (6) 6.5% (3) 4.3% (2)	77.8% (14) 16.7% (3) 5.6% (1) 5.6% (1)	0.60

### Patient-reported outcomes: KOOS-questionnaire and EQ-5D-5L

The outcomes of the KOOS-questionnaire and the EQ-5D-5L from the participants compared to a matched population group are represented in Table 2. For the KOOS-questionnaire, all categories showed significantly lower outcomes when comparing patients who sustained a tibial plateau fracture to a matched population with no reported knee ailments. Next to worse knee-related outcomes, the quality of life, as indicated by the EQ-5D-5L index score, was also lower for patients with tibial plateau fractures compared to a matched sample from the healthy population. For the Overall Health Score, there were no significant differences between both groups.

**Table 2:** KOOS and EQ-5D-5L compared to matched healthy population.

	Participants (n = 46)	Matched healthy population (n = 46)	P value
**KOOS**			
Symptoms*	72.6 ± 21.7	92.5 ± 2.7	<0.001^*ꝉ*^
Pain*	63.9 ± 32.9	93.0 ± 2.5	<0.001^*ꝉ*^
Activities of daily living*	77.4 ± 22.3	94.7 ± 2.7	<0.001^*ꝉ*^
Sport & recreation function*	55.7 ± 35.5	83.5 ± 6.1	<0.001^*ꝉ*^
Knee-related quality of life*	51.9 ± 31.8	90.0 ± 4.1	<0.001^*ꝉ*^
**EQ-5D-5L**			
Index value*	0.79 ± 0.26	0.88 ± 0.03	0.02^*ꝉ*^
Overall Health Score (VAS scale 0–100)*	77.1 ± 17.5	78.0 ± 1.7	0.7^*ꝉ*^

*The values are given as the mean and standard deviation

^*ꝉ*^ *Student t test.*

Differences between men and women in patient-reported outcomes were analyzed within our group; comparing our patient population to a matched population (Table 3). In our study, for both men and women who suffered from a tibial plateau fracture; symptoms, pain, activities of daily living, sport and recreation function, and knee-related quality of life were all significantly worse than those of the matched healthy population. For the overall quality of life, assessed by the EQ-5D-5L questionnaire, men showed significant lower index values compared to the matched healthy population. When comparing the index value of men compared to women, men showed a significant lower index value compared to women. However, no significant differences were observed for the Overall Health Scores. In women, neither the index value nor the Overall Health Score significantly differed between patients who suffered from a tibial plateau fracture and the matched healthy population.

**Table 3:** KOOS and EQ-5D-5L compared to matched healthy population; differences between men and woman.

	Men (n=20)	Matched Healthy population - Men (n=20)	P value	Woman (n=26)	Matched Healthy population – woman (n=26)	P value
**KOOS-questionnaire**						
Symptoms*	67.6±27.0	93.5±2.5	<0.001	76.4±16.8	91.8±2.7	<0.001*^ꝉ^*
Pain*	55.0±37.6	93.6±2.1	<0.001	70.3±28.0	92.5±2.7	<0.001*^ꝉ^*
Activities of daily living*	72.1±26.8	95.8±2.7	<0.001	81.5±17.6	93.8±2.3	<0.001*^ꝉ^*
Sport & recreation function*	48.5±38.1	88.2±5.0	<0.001	61.5±32.9	79.9±4.2	0.007*^ꝉ^*
Knee-related quality of life*	49.7±35.9	89.9±3.1	<0.001	53.7±28.8	90.1 ±4.7	<0.001*^ꝉ^*
**EQ-5D-5L**						
Index value*	0.69±0.36	0.90±0.02	0.01	0.87±0.13	0.88±0.09	0.79*^ꝉ^*
Index value Men vs Woman	0.69±0.36			0.87±0.13		0.02*^ꝉ^*
Overall Health Score (VAS scale 0-100)*	73.4±20.9	78.5±0.9	0.3	81.4±17.1	81.3±13.5	0.4*^ꝉ^*

*The values are given as the mean and standard deviation

^*ꝉ*^ *Student t test.*

### Patient experiences

Based on the results of the outcomes that were observed in our returned questionnaires and the comparison to a matched healthy population, a question arose regarding the factors contributing to worse knee-related outcomes and overall quality of life. Presumably, the primary contributing factor is likely posttraumatic osteoarthritis (PTOA) following a tibial plateau fracture. However, there might be more contributing factors for their recovery and quality of life that are unrelated to PTOA. Consequently, we collected additional data through using semi-structured interviews. Data saturation occurred after conducting 12 patient interviews. However, after the initial round of interviews, the mean age was 66,2 years old, which was relatively advanced compared to the mean age (52.8 years) of the patients included in the first part of our study. To account for potential variations, we decided to conduct six more interviews to include more participants of a younger age as the impact of the working patient group could be different. This resulted in a total of 18 of the previous included patients consented for an interview regarding their treatment after they suffered from a tibial plateau fracture. This selected group represents different age groups; from young people who were working full time to mid-aged people and retirees (Table 1). Of this group, 67% was female and 33% was male and the mean age was 60 years (range 27–81 years).

The structured interview was categorized into four main categories and one of the categories was split up into four subcategories and examples of the participants’ quotes for each category are presented in Table 4.

**Table 4:** Examples of participant quotes from each category assessed during the interview.

Category	Participant quotes
(In) Hospital Experiences	‘It was horrifying that it took 3 days to get to theatre for the surgery. This part I would scale it as a 5. Because I was just waiting and uninformed. If I would have known that I had to wait 3 days for surgery in advance, I would have used her private health care earlier.’
	‘… the only problem with the hospital stay was the pain relief. The nurses were really busy, so I had to wait really long sometimes for pain relief, so I would score it 7/10’
	‘The treatment process was a 11/10. Just think about it: how incredible is it that I’m able to walk again.’
	‘I would rate it a 9 out of 10, I am very happy and grateful for the care I’ve received. My doctor came in every day to follow up how it was going, I was very satisfied with that.’
Decision making and treatment goals	‘Treatment goals were pretty clear, they explained everything well and felt very included in the decisions. They made very clear to me and my wife that I might never walk again, which makes me more grateful that I am able to do this now.’
	‘I wasn’t properly informed or given any choice regarding the treatment. It would have been great if the doctors explained more. There was little to no communication with the doctors when I was in the hospital. I had difficulties asking my questions during the ward rounds, because doctors would already be gone to the next patient or the other room.’
	*‘*The treatment was alright, but the explanation was limited. I needed a lot more information to completely understand my choices.’
Follow-up	
Clinical visits	‘I had two follow-up appointments with orthopaedics clinic and that was enough for me.’
	‘Follow-up was done via another hospital. I wasn’t happy with this. My caregiver wanted me to do dry-needling, but I felt like I needed a treatment that could help me build my muscles back. So this wasn’t very fit-to-patient. But I couldn’t communicate this back to my doctor, because she wasn’t in my care anymore. I would have liked more follow-up.’
	‘Follow-up was none, visiting the clinic was way down the track.’
Rehabilitation	‘I had hydrotherapy 2-3 times a week. This was quite extensive, but I was happy about this, because it got me moving again.’
	‘I was transferred to McLaren’s vale hospital first, nurses treated me like my mum did. They really felt like family and were very caring for me. … After that I went to a rehabilitation center to learn to walk again. All staff in rehabilitation were 10/10.’
	‘They offered me to go to a rehabilitation center, but I was just so desperate to go home. This was not because of the hospital, but just me wanting to go out of there. So 4 days after surgery I went home, which was to soon in hindsight, since my pain was not under control at all. There was also some confusion about whether I could walk or not. The pain was the worst I think, luckily, I had a good GP who helped me out.’
Self-care	After I was discharged I didn’t have a shower for 10 weeks, since that was upstairs. I couldn’t go grocery shopping either. I fell in between two systems I think; I was too young for senior support and to wealthy for social support. Fortunately I got some help from friends and family, otherwise I wouldn’t have managed.
	When I went home, I was still in a wheelchair. Nothing was arranged, no shower chair etc. nothing was thought about when I got discharged home
	‘… I also would have wanted more support from the hospital because at that time I was taking care of my husband and my son, which was an almost impossible situation at that time. I couldn’t drive, couldn’t go around the house.’
Physical therapy	‘I had nothing like physiotherapy. It kept going in a downwards circle for me.’
	‘… I didn’t get any physio referral, I needed to get around on her own in a wheelchair and couldn’t get out. I think physical therapy could’ve supported with my mobility.’
	‘I would have benefitted from physiotherapy and more follow-up to know whether my knee was healing.’
Recommendations regarding treatment and follow-up	‘Extra follow-up would have been great at 6 months and 1 year. A phone call would then suffice already, but I would have been more reassured about the whole process.’
	‘I would advise to actively refer to physical therapy. I had a foot drop and if I wouldn’t have asked for physio myself, this might have been worse. Therefore, I would certainly recommend physio referral for all patients.’
	‘I would really advise them to take people’s personal living situations into account. For, they didn’t account for my living situation at all. I was living on my own and I would have liked some more help once I got out of hospital. No one told me how I was going to feed, wash and take care of myself and no one helped with that. They expected me to attend follow-up appointments, but how do I get there? The only other options were private healthcare providers that I couldn’t afford. So I therefore felt quite abandoned once I left hospital the first 3 months. Even some phone calls from the hospital to inquire how I was doing would’ve been nice for some comfort.’
	‘I would recommend to have a follow-up appointment after 3 & 6 months to know how the knee is doing. The bone healed, but I wasn’t sure if the ligaments did. It would have been nice if an x-ray would have been made to reassure that everything is healing properly.’
	‘I would advise to actively refer to physical therapy. I dad a foot drop and if I wouldn’t have asked for physio myself, this might have been worse. Therefore, I would certainly recommend physio referral for all patients.’

* S1 Table provides an overview of the KOOS and EQ-5D-5L questionnaires of the participants.

#### 1. (in)Hospital experiences.

Patients reported highly variable experiences during hospital admission, ranging from high satisfaction to notable dissatisfaction. Overall, positive experiences were mainly driven by gratitude toward healthcare staff and a perceived successful treatment outcome, whereas negative experiences were predominantly related to waiting times and perceived limitations in nursing availability and responsiveness.

‘The treatment process was a 11/10. Just think about it: how incredible is that I’m able to walk again.’

In contrast, dissatisfaction mostly related to delays in receiving surgical treatment and difficulties accessing timely care and pain management:

‘It was horrifying that it took 3 days to get to theatre for the surgery.’

‘The nurses were really busy, so I had to wait really long sometimes for pain relief’

#### 2. Decision making and the treatment goals.

Experiences regarding shared decision-making and treatment goals were mixed. While many patients reported clear communication and felt involved in treatment decisions, others described insufficient information and limited opportunity for dialogue with treating physicians during hospitalization.

Several participants described feeling well informed and involved in the decision-making process, with clearly communicated treatment expectations:

‘They explained everything well and made me feel very included in the decisions.’

In contrast, other participants reported limited communication and a lack of involvement in treatment decisions, particularly during ward rounds and surgical decision-making:

‘There was little to no communication with the doctors when I was in the hospital.’

‘I needed a lot more information to completely understand my choices.’

#### 3. Follow-up.

Patients described heterogeneous experiences during the post-discharge period, encompassing follow-up care, rehabilitation, self-care capacity, and access to physiotherapy. Overall, experiences were shaped by the degree of structured support received after discharge.

Most participants considered the number of follow-up visits sufficient, although some expressed a need for additional reassurance during recovery. Rehabilitation experiences were generally positive when structured programs such as hydrotherapy or rehabilitation center admission were provided. However, several participants highlighted major challenges in managing self-care at home after discharge, particularly due to limited practical support and difficulties adapting to daily activities. In addition, a lack of routine referral to physiotherapy was frequently reported and perceived as a barrier to recovery.

‘I would have wanted more support from the hospital because at that time I was taking care of my husband and my son, which was an almost impossible situation.’

‘I had nothing like physiotherapy. It kept going in a downward circle for me.’

#### 4. Recommendations by patients.

When asked to provide recommendations for improving care, participants most emphasized the need for more consistent physiotherapy referral, improved follow-up, and greater consideration of patients’ individual home situations after discharge.

Several participants recommended routine access to physiotherapy and closer multidisciplinary collaboration to support functional recovery:

‘I would advise to actively refer to physical therapy… I would certainly recommend physio referral for all patients.’

Others highlighted the importance of increased follow-up and post-discharge support, particularly for patients with limited home support or complex living situations, including occasional suggestions for post-discharge contact to provide reassurance:

‘I would really advise them to take people’s personal living situations into account… I felt quite abandoned once I left hospital.’

## Discussion

This study aimed to conduct a comprehensive assessment of the long-term outcomes of patients after sustaining a tibial plateau fracture by integrating patient-reported outcomes with qualitative patient experiences and comparing results to a matched healthy population. Overall, patients demonstrated significantly worse knee-related function and quality of life 5–10 years after injury, while qualitative findings highlighted substantial variability in hospital experiences and post-discharge support needs, particularly regarding communication, rehabilitation, and self-care.

### Interpretation of quantitative results

Patients with tibial plateau fractures showed significantly poorer outcomes across all KOOS domains compared with a matched healthy population, indicating persistent long-term impairment in symptoms, pain, activities of daily living, sport and recreation, and knee-related quality of life. Similarly, EQ-5D-5L index scores demonstrated reduced overall health-related quality of life in the fracture cohort.

To investigate potential gender differences in patient-reported outcomes, the studies conducted by Marot et al. [21] and McCaffrey et al. [22] reported on gender disparities in knee-related outcomes and general quality of life. Both studies show significantly worse knee-related patient reported outcomes and general quality of life for woman compared to men. Our analysis showed that both men and women experienced substantially worse KOOS outcomes compared to their respective matched reference. When considering the overall quality of life assessed by the EQ-5D-5L index score, men showed significantly lower values compared to the matched healthy population and compared to women. However, given the small subgroup sizes, these sex-specific findings should be interpreted with caution.

In our study, the conversion rate to a Total Knee Arthoplasty (TKA) was 6.5%. Literature states the conversion rate after suffering from a tibial plateau fracture is widely varying from 2.4–7.3% [26–28]. For elderly patients with tibial plateau fractures, literature suggests fracture treatment with a TKA [6,29–31]. They suggest that the results of a primary TKA are better compared to secondary TKA following primary Open Reduction Internal Fixation (ORIF) in this group. These results are in line with other fractures regarding primary arthroplasty for periarticular fractures [32]. However, functional outcomes in this specific subgroup of patients still require further studies.

### Interpretation of qualitative results

The qualitative analysis revealed three main domains of patient experience: acute hospital care, post-discharge support, and rehabilitation access. Experiences during hospitalization ranged from high satisfaction and gratitude toward staff to dissatisfaction related to waiting times and perceived communication gaps.

Post-discharge care emerged as a key area of concern. While follow-up appointments were generally considered adequate, some patients desired more reassurance during recovery. Many participants reported significant challenges with self-care after discharge, particularly in relation to mobility limitations and insufficient practical support at home. Rehabilitation experiences were mixed, with structured programs such as hydrotherapy or inpatient rehabilitation perceived positively, while others described difficulties when discharged home early.

Access to physiotherapy was frequently highlighted as inconsistent and, in some cases, absent. Patients perceived this as a barrier to recovery and mobility, although these findings reflect subjective experiences and do not allow conclusions regarding the effectiveness of structured physiotherapy pathways.

Overall, this qualitative analysis highlighted the varying experiences and perspectives of patients who underwent treatment for tibial plateau fractures. The findings shed light on areas of improvement for communication, follow-up procedures, particularly a referral to physical therapy, and support services to enhance patient outcomes and satisfaction. These findings are in line with literature on the topic of patient-centered care. The systematic review of McMillan et al. [33] concluded that patients reported a greater sense of quality care when there was improved communication, a trusting relationship, and active engagement between the healthcare provider and the patient. While patient-centered care is a great topic of interest in literature and is being recognized as an effective approach to enhancing healthcare services, the application and implementation of patient-centered care has yet to be implemented into the surgical clinical practice [34,35].

### Study limitations

This study has a couple of limitations. Firstly, our study had a small study sample due to a response rate of 28%. Our study sample of 46 participants, however, was representative for all eligible patients treated for a tibial plateau fracture in our hospital. The limited response rate might be attributed to the long-term follow-up of this study. Out of the 119 patients we contacted, several patients were unreachable due address and telephone number changes. It is worth noting that addresses and phone numbers might have been incorrect due to the transition to an electronic patient file a couple years ago. Nevertheless, it is important to note that this study was conducted at a single trauma center in Australia, which might limit the generalizability of the findings to other healthcare settings on different continents.

Second, a potential limitation of this study is the possibility of selection and response bias. Although responders and non-responders were comparable with respect to age and sex, data on other potentially relevant factors, such as fracture severity, fracture classification, treatment characteristics, concomitant injuries, and follow-up course, were not available for comparison. Therefore, we cannot exclude the possibility that differences in these variables may have influenced study participation and affected the generalizability of our findings.

Third, the results of this study were relying on patient recall for self-reported data, which may introduce recall bias. Patients’ recollections of their experiences 5–10 year after suffering from a tibial plateau fracture may be over- or underestimated. Especially dissatisfaction or ongoing functional limitations could have shaped how earlier care was perceived and reported in this study. Despite the passage of an average of 7.4 years during the follow-up period, most interviewees were able to vividly recall their treatment process, which could be due to the significant impact the fracture and subsequent treatment had on their lives, as shown by the results of this study.

### Clinical implications

These findings indicate that tibial plateau fractures have a significant impact on knee-related outcomes and quality of life for both men and women, 5–10 years after sustaining a tibial plateau fracture, when compared to a matched healthy population. Patient experiences highlight important opportunities for improvement in communication, post-discharge support, and rehabilitation pathways. Future studies should further investigate how structured multidisciplinary care pathways may improve both short- and long-term outcomes.

## Supporting information

S1 Appendix Structured interview guide. (DOCX)

S1 Table Overview of the KOOS and EQ-5D-5L questionnaires of the participants mentioned in Table 4. (DOCX)

S1 File Supporting information: Database longterm outcomes TPF KOOS EQ5D5L participants. (SAV)
