# Supplementary material for: Long-term patient-reported knee-related outcomes and quality of life of patients with tibial plateau fractures: A mixed-methods study
Source: PLoS One. 2026 Aug 28;21(8):e0356057. doi: 10.1371/journal.pone.0356057 (PMC13524282; doi:10.1371/journal.pone.0356057)
Supplement: Supplementary file 1 [file pone.0356057.s001.docx]

**Interview Guide**

**Key themes:**

1. **Preoperative inhospital experiences**

On a scale from 1-10, how satisfied were you with your received care after the break of your knee?

2. **Expectations regarding decision making and treatment goals**

Was it clear what the treatment goals were and did they meet your expectations?

On a scale from 1-10, how involved did you feel in the decision making after the break of your knee?

3. **Postoperative inhospital experiences**

Were you satisfied with the support you’ve received during your hospital stay?

If yes, what caused you to be satisfied?

If no, what would you have wanted differently in your care?

4. **Experiences regarding follow-up, rehabilitation and physical therapy**

Were you satisfied with the amount of follow-up you’ve received after you were discharged from the hospital?

Yes, what caused you to be satisfied?

No, what would you have wanted differently in your care?

5. **Recommendations regarding treatment and follow-up**

What do you think is important for the treatment and follow-up for patients with knee fractures?
