# Supplementary material for: Long-term patient-reported knee-related outcomes and quality of life of patients with tibial plateau fractures: A mixed-methods study
Source: PLoS One. 2026 Aug 28;21(8):e0356057. doi: 10.1371/journal.pone.0356057 (PMC13524282; doi:10.1371/journal.pone.0356057)
Supplement: Supplementary file 2 [file pone.0356057.s002.docx]

|  | Symptoms_KOOS | Pain_KOOS | ADL_KOOS | Sports_  KOOS | QoL_KOOS | EQ-5D-5L |
| --- | --- | --- | --- | --- | --- | --- |
| 68yoF | 96,43 | 83,33 | 98,53 | 100 | 81,25 | 11121 |
| 58YoF | 82,14 | 61,11 | 82,35 | 66,67 | 62,5 | 21333 |
| 72yoF | 78,57 | 86,11 | 85,29 | 45 | 50 | 11221 |
| 31yoM | 78,57 | 88,89 | 91,18 | 65 | 68,75 | 11221 |
| 51YoM | 35,71 | 16,67 | 41,18 | 0 | 6,25 | 32343 |
| 61yoF | 60,71 | 100 | 76,47 | 50 | 31,25 | 21221 |
| 57yoM | 35,71 | 2,78 | 45,31 | 15 | 18,75 | 33333 |
| 50yoF | 71,43 | 47,22 | 66,18 | 55 | 31,25 | 21231 |
| 62yoF | 78,57 | 100 | 97,06 |  | 25 | 11121 |
| 34yoM | 78,57 | 75 | 89,71 | 55 | 56,25 | 0 |
| 66yoF | 46,43 | 19,44 | 62,5 | 45 | 25 | 31333 |
| 49yoF | 78,57 | 83,33 | 100 | 75 | 81,25 | 11121 |
| 55yoF | 100 | 100 | 100 | 100 | 93,75 | 11111 |

**SUPPLEMENTARY TABLE 1:** Overview of the KOOS and EQ-5D-5L questionnaires of the participants mentioned in table 4.
