# Supplementary material for: Long-term patient-reported knee-related outcomes and quality of life of patients with tibial plateau fractures: A mixed-methods study
Source: PLoS One. 2026 Aug 28;21(8):e0356057. doi: 10.1371/journal.pone.0356057 (PMC13524282; doi:10.1371/journal.pone.0356057)
Supplement: Supplementary file 3 [file pone.0356057.s004.docx]

Perhaps, you could consider the comments that have been mentioned and then just put a couple of lines in the discussion to say that they may be options for further work etc? / you have considered them etc.

| Journal requirements | Authors response |
| --- | --- |
| 1. Please ensure that your manuscript meets PLOS ONE's style requirements, including those for file naming. | The authors have re-read the PLOS ONE style requirements and have adjusted the headings and subheadings in this paper. |
| 2. Please include a complete copy of PLOS’ questionnaire on inclusivity in global research in your revised manuscript. Our policy for research in this area aims to improve transparency in the reporting of research performed outside of researchers’ own country or community. The policy applies to researchers who have travelled to a different country to conduct research, research with Indigenous populations or their lands, and research on cultural artefacts. The questionnaire can also be requested at the journal’s discretion for any other submissions, even if these conditions are not met.  Please find more information on the policy and a link to download a blank copy of the questionnaire here: <https://journals.plos.org/plosone/s/best-practices-in-research-reporting>. Please upload a completed version of your questionnaire as Supporting Information when you resubmit your manuscript. | The PLOS’ questionnaire on inclusivity in global research has been completed and submitted with the rebuttal. |
| 3. We note that you have indicated that there are restrictions to data sharing for this study. PLOS only allows data to be available upon request if there are legal or ethical restrictions on sharing data publicly. For more information on unacceptable data access restrictions, please see <http://journals.plos.org/plosone/s/data-availability#loc-unacceptable-data-access-restrictions>. | A data repository has been opened to provide access to the data of this study.  ([ru.rumc.datatpf_t0000748a_dsc_658](https://data.ru.nl/collections/ru/rumc/datatpf_t0000748a_dsc_658)) |
| 4. Please include captions for your Supporting Information files at the end of your manuscript, and update any in-text citations to match accordingly. Please see our Supporting Information guidelines for more information: <http://journals.plos.org/plosone/s/supporting-information>. | We have added captions for all Supporting Information files at the end of the manuscript and ensured that all in-text citations have been updated to match the corresponding Supporting Information labels, in accordance with the journal’s guidelines. |
| 5. We note that there is identifying data in the Supporting Information file Supplementary_table_1_Longterm_outcomes_TPF. Due to the inclusion of these potentially identifying data, we have removed this file from your file inventory. Prior to sharing human research participant data, authors should consult with an ethics committee to ensure data are shared in accordance with participant consent and all applicable local laws. | We have removed the specific ages and adjusted the age to whole numbers in table 4 and supplementary table 1 according to your valuable suggestion. |
| 6. If the reviewer comments include a recommendation to cite specific previously published works, please review and evaluate these publications to determine whether they are relevant and should be cited. There is no requirement to cite these works unless the editor has indicated otherwise. | We carefully reviewed the reviewer comments to determine whether any recommended references were relevant to our manuscript. As no specific publications were recommended for citation, and we did not identify additional references that would substantially strengthen or alter the interpretation of our findings, no new citations have been added to the revised manuscript. |
| Reviewers comments | Authors response |
| Reviewer #1: The presented study shows limitations, as allready confirmed by the authors. The Reponse Rate with 28% is very low. The additional interviews are more related to doctor, nursery and physiotherapy issues and not to technical operating procedures influencing the outcome of tibiaplateau fractures. For this reason, I assume this manuscript is not primarely suitable for PLOS One, but more for a rehabilitation journal. | Dear reviewer #1,  We thank you for carefully evaluating our manuscript and for your comments.  We acknowledge the concerns regarding the response rate (28%) and agree that this represents a limitation of the study. This has already been addressed in the manuscript under the limitations section, where we discuss potential response bias and its implications for interpretation of the findings.  Regarding the comment on the content of the interviews, we respectfully note that the aim of the qualitative component was to capture a broad spectrum of perioperative and post-treatment experiences following tibial plateau fractures, including rehabilitation-related aspects as well as patient-perceived influences on overall outcomes. While some themes indeed relate to rehabilitation, physiotherapy, and nursing care, we believe these perspectives are relevant to understanding the full patient pathway and recovery process, which extends beyond strictly technical surgical considerations. |
| Reviewer #2: Thank you for the opportunity to review this manuscript. The study addresses an important topic and the mixed-methods approach provides a valuable patient-centered perspective on long-term outcomes following tibial plateau fractures. Long-term follow-up and integration of qualitative patient experiences provide meaningful clinical relevance. | Dear reviewer #2,  We sincerely thank you for the positive and constructive evaluation of our manuscript.  We greatly appreciate the recognition of the importance of the study topic, as well as the value of the mixed-methods design in providing a patient-centered perspective on long-term outcomes following tibial plateau fractures. We are very pleased that you found the long-term follow-up and integration of qualitative patient experiences to be of meaningful clinical relevance.  We have read your comments and will address your suggestions below: |
| Methods:  1) The methods section shows a possible selection/response bias which should be further addressed in the manuscript. Only sex and age are compared between responders and non-responders. However, other clinically relevant variables (injury severity/treatment/follow up?/additional injuries/fracture classification) may also have influenced participation and should be addressed if available. If this information is not available this needs to be discussed in the limitation section. | We have added the following sentence to our discussion:  ‘A potential limitation of this study is the possibility of selection and response bias. While responders and non-responders were comparable regarding age and sex, we were unable to compare other potentially relevant clinical characteristics, including fracture severity, fracture classification, treatment characteristics, concomitant injuries, and follow-up, because these data were unavailable. Therefore, we cannot exclude the possibility that differences in these variables may have influenced study participation and affected the generalizability of our findings.’ |
| 2) Were the interviews transcribed manually during the interview? It remains unclear whether interviews were audio-recorded and transcribed verbatim. If not, this could indicate a methodological weakness and needs further explanation as it could lead to loss of information and a biased documentation. | We have adjusted this section according to your suggestion:  ‘The interviews were conducted by one researcher (NG), while a second researcher simultaneously transcribed the interview in real time. After completion, all transcripts were reviewed, verified, and refined for accuracy by the research team.’ |
| 3) The interviewed subgroup consisted predominantly of older female participants. although there is no statistically significant demographic difference between the interview subgroup and the overall cohort, the final sample might still not fully reflect the experiences of younger working age patients, potentially influencing generalizability of qualitative findings - this should be acknowledged in the discussion | Your suggestion has been addressed in our results section, subsection ‘patient experiences’  We have added an extra sentence to the methods’ section for further clarification:  ‘Because the initial interview cohort was older than the overall study population (mean age 66.2 vs. 52.8 years), six additional interviews were conducted to include younger participants and better capture potentially different experiences among working-age patients, resulting in a total of 18 interviews.’ |
| 4) The authors provide some fracture pattern and treatment data - nevertheless, tibial plateau fractures represent a heterogeneous injury entity, and additional characterization regarding fracture severity and classifications, treatment characteristics, complications or residual malalignment may further strengthen interpretation of the reported outcomes. | We thank you for this suggestion and we agree that tibial plateau fractures represent a heterogeneous injury entity, and that fracture severity, classification, and treatment characteristics may influence these long-term outcomes.  To provide relevant clinical context, we extracted and reported key injury characteristics that we considered most suitable to the study objectives, i.e. the presence of multi-trauma and whether fractures were unicondylar or bicondylar and the number of reoperations.  While more detailed subgroup analyses based on fracture classifications (e.g., Schatzker classification) and specific treatment modalities would be of interest, the relatively small sample size limited the feasibility of such analyses and precluded drawing meaningful conclusions from comparisons between individual fracture types or treatment groups |
| 5) The methodology used for comparison with the matched healthy population remains unclear and requires further clarification. Based on the cited references, the comparison group appears to consist of published normative reference values rather than an individually matched control cohort. The term " matched population" might thus be misleading. The rationale for the selected statistical approach should be explained in greater detail, particularly regarding the matching procedure and the rationale for the use of Student´s t-test in this setting. | We thank you for this comment and now reconsidering, we see that we haven’t specified this strategy properly in our methods’ section.  The EQ-5D-5L reference data (from reference 24) provide mean values of the EQ-5D-5L subcategories stratified by age groups. Based on these predefined age categories, a “matched” comparison group was constructed using group-level matching. Accordingly, our study cohort of 46 participants was compared with an age-matched reference population derived from these published normative data. This approach allowed us to contextualize our findings relative to expected health status values within comparable age strata.  We have added the following sentence to our methods section:  ‘Lastly, KOOS and EQ-5D-5L outcomes from a general population were obtained from published normative datasets stratified by age and gender [24, 25]. Based on these strata, a group-level matched reference population was constructed by aligning our patient cohort with the corresponding age- and sex-specific categories. This approach enabled comparison of our study population with an age- and sex-comparable general population without reported knee ailments[24, 25].’ |
| The results section offers valuable insights regarding patients experiences. However, for publication the section would surely benefit from a thorough restructuring including a condensation of the qualitative results. In the present form, a lot of patient quotes are included, but the analytical synthesis of the quoted experiences remains limited. | We agree that the qualitative Results section could benefit from further structuring and condensation, particularly regarding the balance between participant quotations and analytical synthesis.  In response, we have thoroughly revised the Results section. Specifically, we have streamlined the presentation of themes, reduced and selectively retained illustrative quotations, and strengthened the analytical synthesis within each category. This restructuring has improved the clarity and interpretability of the qualitative findings while maintaining the integrity of participants’ perspectives.  We believe these changes have significantly enhanced the readability and scientific quality of the Results section. |
| Discussion: Given the small subgroup sizes, sex-specific comparisons should be interpreted with caution, as the present study is likely underpowered for such analyses.  The discussion would likely benefit from focusing more on interpretation of the results and implications for patient-centered trauma care.  Some of the conclusions appear stronger than supported by the provided data. In particular, the presented data report patient perceptions regarding rehabilitation needs, but the study design does not allow conclusions regarding rhe effectiveness of standardized physiotherapy referral pathways - caution must be taken to not overinterpret exploratory findings.  Recall bias should be discussed in greater detail, particularly given the long follow-up period and the retrospective assessment of patient experiences. Current dissatisfatction may shape retrospective interpretation of past care experiences. | We thank you for your suggestions. We have addressed your concerns and made substantial revisions to the Discussion accordingly:   1. We agree that sex-specific subgroup analyses should be interpreted with caution due to the limited sample size of the subgroups. We have therefore clarified in the discussion that these analyses are exploratory in nature and should be interpreted accordingly. 2. We have adjusted our conclusions regarding the effectiveness of standardized physiotherapy referral. 3. We have elaborated on the topic of recall bias. |
| Others: In general, the results and discussion section are repetitive and should be shortened to better fit the structure and style of an academic journal article. | We have carefully revised both sections. We have streamlined descriptive reporting, removed repetitive statements between sections, and strengthened the distinction between presentation of findings and the interpretation. |
| Several findings indicate that the manuscript would profit from stylistic and grammatical revision by a native speaker, e.g.: Line 67: "patients were sent" instead of "send" Line 79 "raging" instead of "ranging" | The identified errors (including “patients were sent” instead of “send” and “ranging” instead of “raging”) have been corrected, and the manuscript has been thoroughly revised to improve clarity, grammar, and overall language quality. |
|  | Dear reviewer #2,  Thank you for your elaborate review on our paper and your valuable suggestions. We have thoroughly revised our results and discussion section. We believe that our manuscript has improved significantly due to these revisions.  We hope you agree with the changes and that you can support the approval of our manuscript for publication. |
|  |  |
