# Supplementary material for: Long-term patient-reported knee-related outcomes and quality of life of patients with tibial plateau fractures: A mixed-methods study
Source: PLoS One. 2026 Aug 28;21(8):e0356057. doi: 10.1371/journal.pone.0356057 (PMC13524282; doi:10.1371/journal.pone.0356057)
Supplement: Peer review history

# Peer review: Decision Letter 0

**DOI:** 10.1371/journal.pone.0356057.r001

**Authors:** Hans-Peter Simmen

18 May 2026

Dear Dr. Gaast,

Thank you for submitting your manuscript to PLOS ONE. After careful consideration, we feel that it has merit but does not fully meet PLOS ONE’s publication criteria as it currently stands. Therefore, we invite you to submit a revised version of the manuscript that addresses the points raised during the review process.

Please submit your revised manuscript by end of Jul 02 2026 11:59PM. If you will need more time than this to complete your revisions, please reply to this message or contact the journal office at plosone@plos.org. When you're ready to submit your revision, log on to https://www.editorialmanager.com/pone/ and select the 'Submissions Needing Revision' folder to locate your manuscript file.

- A letter that responds to each point raised by the academic editor and reviewer(s). You should upload this letter as a separate file labeled 'Response to Reviewers'.
- A marked-up copy of your manuscript that highlights changes made to the original version. You should upload this as a separate file labeled 'Revised Manuscript with Track Changes'.
- An unmarked version of your revised paper without tracked changes. You should upload this as a separate file labeled 'Manuscript'.

If you would like to make changes to your financial disclosure, please include your updated statement in your cover letter. Guidelines for resubmitting your figure files are available below the reviewer comments at the end of this letter.

If applicable, we recommend that you deposit your laboratory protocols in protocols.io to enhance the reproducibility of your results. Protocols.io assigns your protocol its own identifier (DOI) so that it can be cited independently in the future. For instructions see: https://journals.plos.org/plosone/s/submission-guidelines#loc-laboratory-protocols. Additionally, PLOS ONE offers an option for publishing peer-reviewed Lab Protocol articles, which describe protocols hosted on protocols.io. Read more information on sharing protocols at https://plos.org/protocols?utm_medium=editorial-email&utm_source=authorletters&utm_campaign=protocols.

As the corresponding author, your ORCID iD is verified in the submission system and will appear in the published article. PLOS supports the use of ORCID, and we encourage all coauthors to register for an ORCID iD and use it as well. Please encourage your coauthors to verify their ORCID iD within the submission system before final acceptance, as unverified ORCID iDs will not appear in the published article. *Only*  the individual author can complete the verification step; PLOS staff *cannot*  verify ORCID iDs on behalf of authors.

We look forward to receiving your revised manuscript.

Kind regards,

Hans-Peter Simmen, M.D., Professor of Surgery

Academic Editor

PLOS One

Journal Requirements:

When submitting your revision, we need you to address these additional requirements.

1. Please ensure that your manuscript meets PLOS ONE's style requirements, including those for file naming. The PLOS ONE style templates can be found at

https://journals.plos.org/plosone/s/file?id=wjVg/PLOSOne_formatting_sample_main_body.pdf and

https://journals.plos.org/plosone/s/file?id=ba62/PLOSOne_formatting_sample_title_authors_affiliations.pdf

2. Please include a complete copy of PLOS’ questionnaire on inclusivity in global research in your revised manuscript. Our policy for research in this area aims to improve transparency in the reporting of research performed outside of researchers’ own country or community. The policy applies to researchers who have travelled to a different country to conduct research, research with Indigenous populations or their lands, and research on cultural artefacts. The questionnaire can also be requested at the journal’s discretion for any other submissions, even if these conditions are not met.  Please find more information on the policy and a link to download a blank copy of the questionnaire here: https://journals.plos.org/plosone/s/best-practices-in-research-reporting. Please upload a completed version of your questionnaire as Supporting Information when you resubmit your manuscript.

3. We note that you have indicated that there are restrictions to data sharing for this study. PLOS only allows data to be available upon request if there are legal or ethical restrictions on sharing data publicly. For more information on unacceptable data access restrictions, please see http://journals.plos.org/plosone/s/data-availability#loc-unacceptable-data-access-restrictions.

Before we proceed with your manuscript, please address the following prompts:

a) If there are ethical or legal restrictions on sharing a de-identified data set, please explain them in detail (e.g., data contain potentially identifying or sensitive patient information, data are owned by a third-party organization, etc.) and who has imposed them (e.g., a Research Ethics Committee or Institutional Review Board, etc.). Please also provide contact information for a data access committee, ethics committee, or other institutional body to which data requests may be sent.

b) If there are no restrictions, please upload the minimal anonymized data set necessary to replicate your study findings to a stable, public repository and provide us with the relevant URLs, DOIs, or accession numbers. For a list of recommended repositories, please see

https://journals.plos.org/plosone/s/recommended-repositories. You also have the option of uploading the data as Supporting Information files, but we would recommend depositing data directly to a data repository if possible.

We will update your Data Availability statement on your behalf to reflect the information you provide.

4. Please include captions for your Supporting Information files at the end of your manuscript, and update any in-text citations to match accordingly. Please see our Supporting Information guidelines for more information: http://journals.plos.org/plosone/s/supporting-information.

5. We note that there is identifying data in the Supporting Information file Supplementary_table_1_Longterm_outcomes_TPF. Due to the inclusion of these potentially identifying data, we have removed this file from your file inventory. Prior to sharing human research participant data, authors should consult with an ethics committee to ensure data are shared in accordance with participant consent and all applicable local laws.

Data sharing should never compromise participant privacy. It is therefore not appropriate to publicly share personally identifiable data on human research participants. The following are examples of data that should not be shared:

-Name, initials, physical address

-Ages more specific than whole numbers

-Internet protocol (IP) address

-Specific dates (birth dates, death dates, examination dates, etc.)

-Contact information such as phone number or email address

-Location data

-ID numbers that seem specific (long numbers, include initials, titled “Hospital ID”) rather than random (small numbers in numerical order)

Data that are not directly identifying may also be inappropriate to share, as in combination they can become identifying. For example, data collected from a small group of participants, vulnerable populations, or private groups should not be shared if they involve indirect identifiers (such as sex, ethnicity, location, etc.) that may risk the identification of study participants.

Additional guidance on preparing raw data for publication can be found in our Data Policy (https://journals.plos.org/plosone/s/data-availability#loc-human-research-participant-data-and-other-sensitive-data) and in the following article: http://www.bmj.com/content/340/bmj.c181.long.

Please remove or anonymize all personal information age, ensure that the data shared are in accordance with participant consent, and re-upload a fully anonymized data set. Please note that spreadsheet columns with personal information must be removed and not hidden as all hidden columns will appear in the published file.

6. If the reviewer comments include a recommendation to cite specific previously published works, please review and evaluate these publications to determine whether they are relevant and should be cited. There is no requirement to cite these works unless the editor has indicated otherwise.

[Note: HTML markup is below. Please do not edit.]

Reviewers' comments:

Reviewer's Responses to Questions

**Comments to the Author**

1. Is the manuscript technically sound, and do the data support the conclusions?

Reviewer #1: Partly

Reviewer #2: Partly

**********

2. Has the statistical analysis been performed appropriately and rigorously?

Reviewer #1: I Don't Know

Reviewer #2: Yes

**********

3. Have the authors made all data underlying the findings in their manuscript fully available?

The PLOS Data policy

Reviewer #1: No

Reviewer #2: Yes

**********

4. Is the manuscript presented in an intelligible fashion and written in standard English?

Reviewer #1: Yes

Reviewer #2: No

**********

Reviewer #1: The presented study shows limitations, as allready confirmed by the authors. The Reponse Rate with 28% is very low.

The additional interviews are more related to doctor, nursery and physiotherapy issues and not to technical operating procedures influencing the outcome of tibiaplateau fractures.

For this reason, I assume this manuscript is not primarely suitable for PLOS One, but more for a rehabilitation journal.

Reviewer #2: Thank you for the opportunity to review this manuscript. The study addresses an important topic and the mixed-methods approach provides a valuable patient-centered perspective on long-term outcomes following tibial plateau fractures. Long-term follow-up and integration of qualitative patient experiences provide meaningful clinical relevance.

I think the authors should address the following methodological and structural aspects before this manuscript can be considered for publication:

Methods:

1) The methods section shows a possible selection/response bias which should be further addressed in the manuscript. Only sex and age are compared between responders and non-responders. However, other clinically relevant variables (injury severity/treatment/follow up?/additional injuries/fracture classification) may also have influenced participation and should be addressed if available. If this information is not available this needs to be discussed in the limitation section.

2) Were the interviews transcribed manually during the interview? It remains unclear whether interviews were audio-recorded and transcribed verbatim. If not, this could indicate a methodological weakness and needs further explanation as it could lead to loss of information and a biased documentation.

3) The interviewed subgroup consisted predominantly of older female participants. although there is no statistically significant demographic difference between the interview subgroup and the overall cohort, the final sample might still not fully reflect the experiences of younger working age patients, potentially influencing generalizability of qualitative findings - this should be acknowledged in the discussion

4) The authors provide some fracture pattern and treatment data - nevertheless, tibial plateau fractures represent a heterogeneous injury entity, and additional characterization regarding fracture severity and classifications, treatment characteristics, complications or residual malalignment may further strengthen interpretation of the reported outcomes.

5) The methodology used for comparison with the matched healthy population remains unclear and requires further clarification. Based on the cited references, the comparison group appears to consist of published normative reference values rather than an individually matched control cohort. The term " matched population" might thus be misleading. The rationale for the selected statistical approach should be explained in greater detail, particularly regarding the matching procedure and the rationale for the use of Student´s t-test in this setting.

Results:

The results section offers valuable insights regarding patients experiences. However, for publication the section would surely benefit from a thorough restructuring including a condensation of the qualitative results. In the present form, a lot of patient quotes are included, but the analytical synthesis of the quoted experiences remains limited.

Discussion:

Given the small subgroup sizes, sex-specific comparisons should be interpreted with caution, as the present study is likely underpowered for such analyses.

The discussion would likely benefit from focusing more on interpretation of the results and implications for patient-centered trauma care.

Some of the conclusions appear stronger than supported by the provided data. In particular, the presented data report patient perceptions regarding rehabilitation needs, but the study design does not allow conclusions regarding rhe effectiveness of standardized physiotherapy referral pathways - caution must be taken to not overinterpret exploratory findings.

Recall bias should be discussed in greater detail, particularly given the long follow-up period and the retrospective assessment of patient experiences. Current dissatisfatction may shape retrospective interpretation of past care experiences.

Others:

In general, the results and discussion section are repetitive and should be shortened to better fit the structure and style of an academic journal article.

Several findings indicate that the manuscript would profit from stylistic and grammatical revision by a native speaker, e.g.:

Line 67: "patients were sent" instead of "send"

Line 79 "raging" instead of "ranging"

**********

what does this mean?). If published, this will include your full peer review and any attached files.

If you choose “no”, your identity will remain anonymous but your review may still be made public.

**Do you want your identity to be public for this peer review?**  For information about this choice, including consent withdrawal, please see our Privacy Policy

Reviewer #1: No

Reviewer #2: No

**********

[NOTE: If reviewer comments were submitted as an attachment file, they will be attached to this email and accessible via the submission site. Please log into your account, locate the manuscript record, and check for the action link "View Attachments". If this link does not appear, there are no attachment files.]

To ensure your figures meet our technical requirements, please review our figure guidelines: https://journals.plos.org/plosone/s/figures

You may also use PLOS’s free figure tool, NAAS, to help you prepare publication quality figures: https://journals.plos.org/plosone/s/figures#loc-tools-for-figure-preparation.

NAAS will assess whether your figures meet our technical requirements by comparing each figure against our figure specifications.

# Author comment: Author response to Decision Letter 1

**DOI:** 10.1371/journal.pone.0356057.r002

16 Jul 2026

Dear Editor,

Thank you for the opportunity to revise and resubmit our manuscript. We sincerely appreciate the time and effort that you and the reviewers have invested in evaluating our work. We have taken the time to carefully consider each of the comments and suggestions provided, and have thoroughly revised the manuscript accordingly. The constructive feedback has been invaluable in improving both the scientific quality and the clarity of our work.

In response to the reviewers' and editorial comments, we have addressed all concerns in the accompanying point-by-point rebuttal. The revisions include substantial improvements to the methodology and discussion, clarification of our study design and statistical approach, a more balanced interpretation of the findings, and a streamlined presentation of the qualitative results. We have also expanded the discussion of the study's limitations, including potential selection and recall bias, and improved the reporting of the interview methodology.

In addition, we have addressed all editorial requirements by ensuring compliance with PLOS ONE formatting guidelines, completing and submitting the inclusivity questionnaire, making the study data publicly available through a data repository, updating the Supporting Information and captions, removing potentially identifiable information, and carefully revising the manuscript for language, grammar, and readability.

We believe that these revisions have significantly strengthened the manuscript and have addressed the concerns raised during the review process. We are grateful for the thoughtful feedback, which has helped improve both the scientific rigor and the presentation of our work.

Thank you for your consideration of our revised manuscript. We hope that the revisions meet the journal's expectations, and we look forward to your further evaluation.

Sincerely,

On behalf of all authors,

Nynke van der Gaast

Attachment Submitted filename: V2_Rebuttal Reviewers longterm outcomes for TPF.docx

# Editor report: Decision Letter 1

**DOI:** 10.1371/journal.pone.0356057.r003

**Authors:** Hans-Peter Simmen

29 Jul 2026

Long-term patient-reported knee-related outcomes and quality of life of patients with tibial plateau fractures; a mixed methods study

PONE-D-26-15870R1

Dear Dr. Gaast,

Congratulations ! After revision your manuscript is much better. We’re pleased to inform you that your manuscript has been judged scientifically suitable for publication and will be formally accepted for publication once it meets all outstanding technical requirements.

Within one week, you’ll receive an e-mail detailing the required amendments. When these have been addressed, you’ll receive a formal acceptance letter and your manuscript will be scheduled for publication.

An invoice will be generated when your article is formally accepted. Please note, if your institution has a publishing partnership with PLOS and your article meets the relevant criteria, all or part of your publication costs will be covered. Please make sure your user information is up-to-date by logging into Editorial Manager at Editorial Manager® and clicking the ‘Update My Information' link at the top of the page. For questions related to billing, please contact billing support.

If your institution or institutions have a press office, please notify them about your upcoming paper to help maximize its impact. If they’ll be preparing press materials, please inform our press team as soon as possible -- no later than 48 hours after receiving the formal acceptance. Your manuscript will remain under strict press embargo until 2 pm Eastern Time on the date of publication. For more information, please contact onepress@plos.org.

Kind regards,

Hans-Peter Simmen, M.D., Professor of Surgery

Academic Editor

PLOS One

Additional Editor Comments (optional):

Reviewers' comments:

# Editor report: Acceptance letter

**DOI:** 10.1371/journal.pone.0356057.r004

**Authors:** Hans-Peter Simmen

PONE-D-26-15870R1

PLOS One

Dear Dr. Gaast,

I'm pleased to inform you that your manuscript has been deemed suitable for publication in PLOS One. Congratulations! Your manuscript is now being handed over to our production team.

At this stage, our production department will prepare your paper for publication. This includes ensuring the following:

* All references, tables, and figures are properly cited

* All relevant supporting information is included in the manuscript submission,

* There are no issues that prevent the paper from being properly typeset

You will receive further instructions from the production team, including instructions on how to review your proof when it is ready. Please keep in mind that we are working through a large volume of accepted articles, so please give us a few days to review your paper and let you know the next and final steps.

Lastly, if your institution or institutions have a press office, please let them know about your upcoming paper now to help maximize its impact. If they'll be preparing press materials, please inform our press team within the next 48 hours. Your manuscript will remain under strict press embargo until 2 pm Eastern Time on the date of publication. For more information, please contact onepress@plos.org.

You will receive an invoice from PLOS for your publication fee after your manuscript has reached the completed accept phase. If you receive an email requesting payment before acceptance or for any other service, this may be a phishing scheme. Learn how to identify phishing emails and protect your accounts at https://explore.plos.org/phishing.

If we can help with anything else, please email us at customercare@plos.org.

Thank you for submitting your work to PLOS One and supporting open access.

Kind regards,

PLOS ONE Editorial Office Staff

on behalf of

Dr. Hans-Peter Simmen

Academic Editor

PLOS One
